# Preliminary study showing no association between G238A (rs361525) tumor necrosis factor-α (TNF-α) gene polymorphism and its serum level, hormonal and biochemical aspects of polycystic ovary syndrome

**DOI:** 10.1186/s12881-018-0662-1

**Published:** 2018-08-22

**Authors:** Fahimeh Kordestani, Sahar Mazloomi, Yousef Mortazavi, Saeideh Mazloomzadeh, Mojtaba Fathi, Haleh Rahmanpour, Abolfazl Nazarian

**Affiliations:** 1 Department of Biochemistry, School of Medicine, Zanjan University of Medical Sciences, Zanjan, Iran; 2 Department of Medical Biotechnology and Nanotechnology, School of Medicine, Zanjan University of Medical Sciences, Zanjan, Iran; 3 Zanjan Metabolic Disease Research Center, Valiasr Hospital, Zanjan University of Medical Science, Zanjan, Iran; 4 Social Determinants of Health Research Center, Zanjan University of Medical Sciences, Zanjan, Iran; 5 Department of Biochemistry, School of Medicine, Zanjan University of Medical Sciences, PO Box: 4513956111, Zanjan, Iran; 6 Department of Obstetrics and Gynecology, School of Medicine, Zanjan University of Medical Science, Zanjan, Iran; 7 Department of Biochemistry and Nutrition, Faculty of Medicine, Zanjan University of Medical Sciences, Zanjan, Iran

**Keywords:** Polycystic ovary syndrome (PCOS), Tumor necrosis factor-alpha (TNF-α), Hormone profile, Polymorphism, Polymerase chain reaction–restriction fragment length polymorphism (PCR-RFLP)

## Abstract

**Background:**

Polycystic ovary syndrome (PCOS) is the main cause of female infertility. Interactions among genetic, biochemical, and immunological factors can affect the pathogenesis of PCOS. As a proinflammatory cytokine, tumor necrosis factor-α (TNF-α) plays an important role in this regard. The present study aimed to evaluate the association of the rs361525 gene single-nucleotide polymorphism (SNP) and TNF-α serum levels with the hormonal and biochemical characteristics of PCOS in Iranian individuals.

**Methods:**

The SNP rs361525 in the *TNF-α* gene was analyzed by polymerase chain reaction–restriction fragment length polymorphism (PCR-RFLP) in a total of 111 PCOS patients and 105 healthy females. Serum levels of TNF-α, lipid and hormone profiles, and biochemical factors were measured using enzyme-linked immunosorbent assay (ELISA) and calorimetric methods, as appropriate.

**Results:**

The TNF-α serum level was higher in women with PCOS compared with the control group (*p* <  0.0001), and it was significantly correlated with the homeostasis model assessment (HOMA) factor (*r* = 0.138, *p* <  0.05). No significant differences were found in the genotype and allelic frequencies between the two groups (*p* >  0.05). Higher levels and significant differences were found for the HOMA factor, luteinizing hormone/follicle-stimulating hormone (LH/FSH), testosterone, and body mass index (BMI) in the PCOS group compared with the control group (*p* <  0.0001). High LH/FSH ratios (odds ratio [OR] = 1.98, 95% confidence interval [CI] = 1.20–3.28, *p* <  0.01), and high HOMA factor (OR = 5.04, 95% CI = 2.82–9.01, *p* <  0.001) were significantly associated with an increased risk of PCOS.

**Conclusions:**

Despite the lack of significant difference between rs361525 polymorphism of the *TNF-α* gene and PCOS, the serum level of TNF-α was increased in PCOS patients and positively correlated with the HOMA factor. Elevation of the LH/FSH ratio and HOMA for insulin resistance (HOMA-IR) increased the risk of PCOS. Therefore, TNF-α could indirectly contribute to PCOS progression.

## Background

Polycystic ovary syndrome (PCOS) is a major cause of female infertility, affecting 6–10% of women during reproductive age; moreover, it is one of the most prevalent endocrine disorders [1]. This syndrome is associated with increased risks of obesity, type 2 diabetes mellitus, hyperinsulinemia, insulin resistance, cardiovascular disease, and dyslipidemia [1, 2]. Evidence indicates an interaction among genetic, biochemical, environmental, and immunological factors in the pathogenesis of PCOS [2, 3] .Among the immunological factors, a disequilibrium of pro−/anti-inflammatory cytokines has been offered as a key contributor [2].

As a proinflammatory cytokine, tumor necrosis factor-α (TNF-α) is secreted by ovarian macrophages, granulose-luteal cells, and immune cells [4]. In addition to interference in immune and inflammation responses, differentiation, proliferation, and cell death [5], TNF-α has a role in PCOS patients with obesity [6], insulin resistance [7, 8], hyperandrogenism [9], and PCOS patients with hyperandrogenism [10]. In contrast, the production of TNF-α in granulosa cells in PCOS patients decreases aromatase gene expression. This process occurs via the inhibition of adenylyl cyclase and the cyclic adenosine monophosphate (cAMP) signaling pathway, resulting in the reduction of 17-β-estradiol production from the ovary; consequently, elevated ovarian androgen is one of the most common characteristic of PCOS patients [11, 12].

TNF-α induces serine phosphorylation in insulin receptor substrate-1 (IRS-1), resulting in the inhibition of tyrosine kinase activity in the insulin receptor and leading to insulin resistance and hyperinsulinemia [8, 10]. This process is also the cause of a low production of sex hormone–binding globulins in the liver, which increases the free androgen serum level [11]. Accordingly, a direct relationship between the serum levels of TNF-α and androgen in PCOS patients has been identified in some studies [13, 14].

TNF-α is encoded by a gene located on chromosome 6p21.3, and it has a promoter of 1100 bp in length. Nucleotide substitution in this region can affect transcription factors’ binding affinity, and subsequently, the level of gene expression. Therefore, different concentrations of serum TNF-α can be produced, leading to many sorts of disorders [3, 15]. Studies in Chinese, Korean, and South Indian populations have revealed a relationship between polymorphisms in the promoter region of the *TNF-α* gene and PCOS [1, 3, 16], hyperandrogenism [9], type 2 diabetes [17], and obesity [18].

In a case-control study of a Korean population, G allele carriers of single-nucleotide polymorphism (SNP) rs361525 in the *TNF-α* gene showed an association with overweight/obesity susceptibility [18]. It has been demonstrated that a G238A *TNF-α* SNP in the promoter region could be associated with diabetes, and the 238A/308G haplotype has been shown to elevate the TNF-α serum level in an Indian population [17]. Overall, based on different studies, it has been shown that *TNF-α* SNPs can elevate the serum levels of TNF-α, and this could be associated with PCOS [13, 19, 20].

In our study, the *TNF-α* G238A SNP (rs361525) was selected based on previous studies showing positive associations between this SNP and serum levels of testosterone and insulin, obesity, and so on which all of them are the properties of PCOS. Therefore, the lack of research regarding the relationship between PCOS and SNP rs361525 in the *TNF-α* gene in the gene databases was the basis for this selection. To the best of our knowledge, this is the first study to investigate the genotyping of rs361525 polymorphism and determination of TNF-α serum levels in Iranian PCOS patients, including an evaluation of the effects of this factor on serum lipid profiles and related endocrine and biochemical factors.

## Methods

### Study population and sample collection

In the present study, a total of 216 women comprising 111 PCOS patients and 105 controls were recruited from the Endocrinology Clinic of Valiasr Hospital and Gynecology Clinic of Mousavi Hospital, Zanjan, Iran. According to the Rotterdam consensus, PCOS is characterized by two out of three of the following: clinical and/or biochemical signs of hyperandrogenism, polycystic ovaries on sonography, and oligo−/anovulation [21]. Patients with inflammatory diseases, acute or chronic infections, Cushing’s syndrome, and androgen-secreting tumors were excluded. The selected participants had not taken any hormonal or anti-inflammatory medicine for 3–6 months before entering the study. All subjects were new cases for PCOS.

This study was approved by ethics committee (No. ZUMS.REC.1394.90) of Zanjan University of Medical Sciences of Iran. Written informed consent was received from all the subjects before blood sampling.

For sample size calculation, we conducted a pilot study including 20 PCOS patients and 20 healthy individuals. After completion of the experiment, 25% of PCOS cases and 10% of healthy control were carrier. Sample size was calculated 100 per group based on P1 = 0.10, P2 = 0.25, α = 0.05, β = 0.20 using the formula of comparing two proportions.

Blood specimens were collected from subjects in two separate tubes on days 3–5 of their menstrual period, following the World Health Organization (WHO) guidelines. Anticoagulated whole blood samples were taken for DNA extraction and serum for biochemical parameters. Serum samples were kept at − 20 °C until determination of the biochemical parameters. Genomic DNA was extracted from white blood cells using the Bioneer genomic DNA extraction kit (Bioneer, Korea, Cat. No. K-3032). The DNA quality was determined with 260/280 optical density (OD) ratios in all samples, which were stored at − 20 °C until use.

### Genotyping

#### Polymerase chain reaction (PCR)

**Fig. 1:**
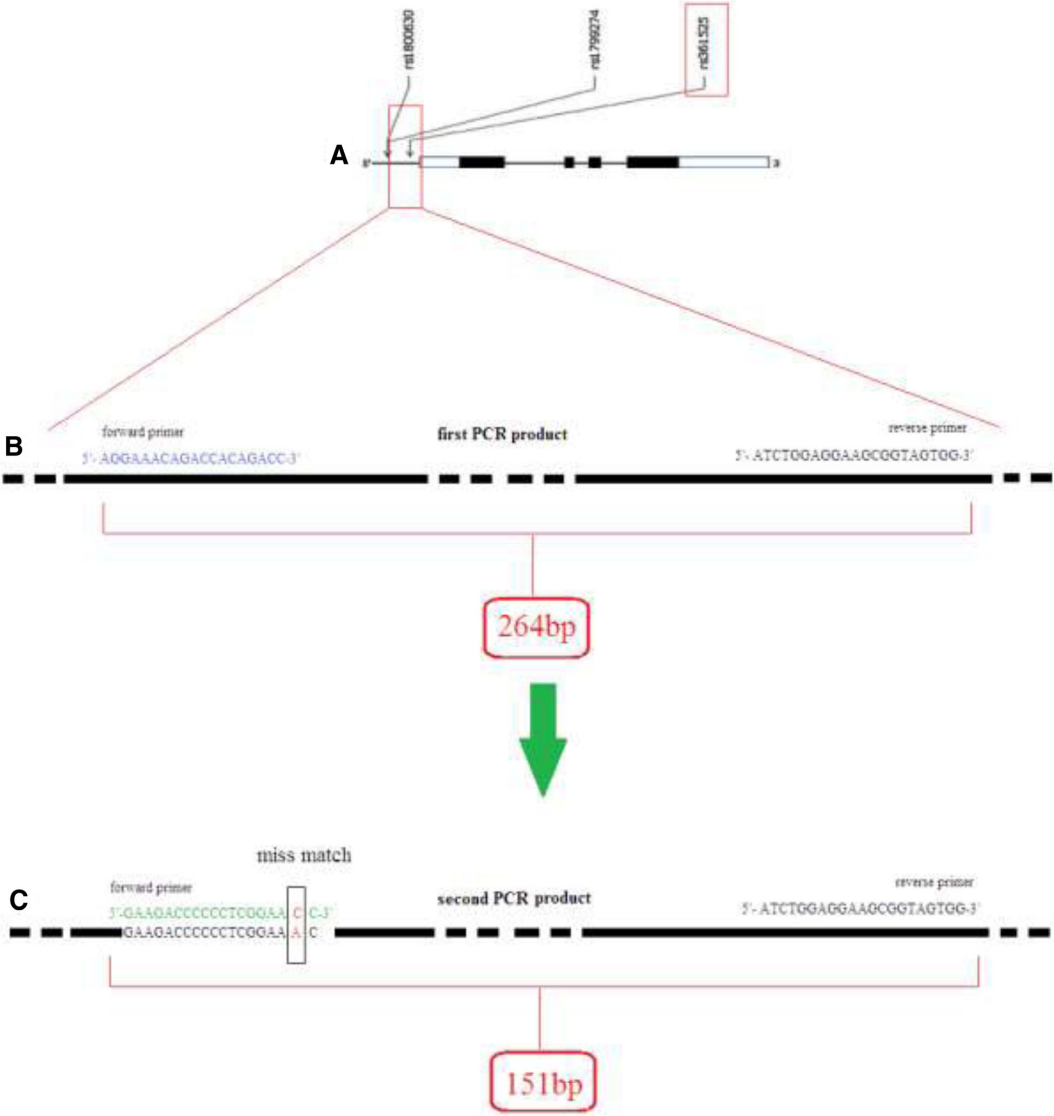
**a** Gene map of a single-nucleotide polymorphism (SNP) in *TNF-α* gene on chromosome 6. Semi-nested PCR with two pairs of primers. The reverse primer was common for both PCRs. **b** Primers and PCR product size in the first PCR. **c** Primers and PCR product size in the second PCR (semi-nested). (TNF-α: tumor necrosis factor-α)

In this study, polymerase chain reaction–restriction fragment length polymorphism (PCR-RFLP) was used for genotyping of G238A (rs361525) in the promoter region of the *TNF-α* gene. Semi-nested PCR was performed using two pairs of primers with the following sequences: forward, 5′-AGGAAACAGACCACAGACC-3′; reverse, 5′-ATCTGGAGGAAGCGGTAGTGG-3′. These were used in the first PCR reaction. The PCR product size was 264 bp. The primers for the second PCR reaction were 5′-GAAGACCCCCCTCGGAACC-3′ (forward) and 5′ATCTGGAGGAAGCGGTAGTGG-3′ (reverse), with a product size of 151 bp. The restriction site was designed to be situated on the forward primer in the second PCR reaction (Fig. 1).

PCR was performed according to the manufacturer’s protocol (Ampliqon, Denmark; PCR Master Mix 2× containing Taq DNA polymerase, buffer, MgCl_2_, and dNTP). DNA template (200 ng) and 10 μmol/L of each primer were added to the PCR reaction mix (25 μL). Amplification was carried out with a thermal cycler (Flex Cycler^2^, Germany) under the following conditions: for the first PCR, an initial denaturation at 95 °C for 5 min, followed by 35 cycles of denaturation at 92 °C for 30 s, annealing at 62 °C for 30 s, and an extension at 72 °C for 45 s. For the second PCR, the initial denaturation took place at 95 °C for 5 min, followed by 40 cycles of denaturation at 92 °C for 30 s, annealing for 63 °C for 30 s, and an extension at 72 °C for 45 s. Finally, both PCRs were followed by a final extension at 72 °C for 5 min. The PCR products were separated using 2.5% agarose (Invitrogen, USA) gel electrophoresis and visualized with an ultraviolet transilluminator after staining with DNA safe stain (EURx, Poland).

#### Restriction fragment length polymorphism (RFLP)

Products of the first PCR were used as a template for the second PCR, so digestion was performed on the products of the second PCR (151 bp) using 0.5 units of *HpaII* restriction endonuclease (CinnaGen, RD1171), following the manufacturer’s recommendations. Briefly, the reaction was incubated at 37 °C for 6 h. Digested fragments were separated on 3% agarose gel by electrophoresis. Fragments of 133 bp and 18 bp were considered to represent the homozygous GG genotype, while segments of 151 bp, 133 bp, and 18 bp represented the heterozygous GA genotype. A single band of a 151-bp fragment was considered a homozygous AA genotype.

### Clinical and biochemical parameter measurement

Waist and hip circumferences, body weight, and height were measured in all subjects as anthropometric variables. The body mass index (BMI) and waist–hip ratio (WHR) were calculated as follows:

BMI = body mass/ (height)^2^[kg/m^2^],

WHR = waist circumference (cm)/hip circumference (cm).

For measurement of the serum levels of follicle-stimulating hormone (FSH; Monobind kit, USA), luteinizing hormone (LH; Monobind kit, USA), testosterone (Monobind kit, USA), estrogen (Monobind kit, USA), insulin (Monobind kit, USA), and TNF-α (eBioscience, Austria), the enzyme-linked immunosorbent assay (ELISA) method was used according to the manufacturer’s recommendations. Color intensities at the final step were recorded using an ELISA reader (Stat Fax-2100 microplate reader, Awareness Technology, USA). The biochemical parameters including fasting blood glucose (Pars azmoon, Iran), triglyceride (Pars azmoon, Iran), total cholesterol (Pars azmoon, Iran), low-density lipoprotein (LDL; Pars azmoon, Iran), and high-density lipoprotein (HDL; Pars azmoon, Iran) were measured using a BT3000 autoanalyzer (Biotechnica Instruments, USA). Homeostasis model assessment (HOMA) as an insulin resistance index was computed using the following formula: HOMA = fasting glucose (mg/dl) × fasting insulin (mU/ml)/405.

### Statistical analysis

All statistical analyses were performed using SPSS 22.0 (Chicago, IL, USA). Data were tested for normal distribution using the Kolmogorov–Smirnov test. Differences between two variables were measured with an independent sample *t*-test for normal distributions, while the Mann–Whitney test was used for non-normally distributed data. The qualitative or quantitative results were expressed as the frequency or mean ± SD, respectively.

The association between groups and biochemical factors were evaluated using regression logistic binary test by calculating the odds ratios (OR) at a 95% CI. Differences in serum levels of FSH, LH, testosterone, estrogen, insulin, TNF-α, fasting blood glucose, triglyceride, total cholesterol, LDL, and HDL between the groups were tested using the independent student *t*-test or Mann–Whitney test, as appropriate. The correlation between continuous variables was assessed using Pearson’s correlation coefficient. A *p*-value less than 0.05 was considered significant.

Differences in the frequency of the alleles and genotypes between the PCOS patients and age-matched healthy subjects were tested using Chi-square tests. The Hardy–Weinberg equilibrium (HWE) was estimated using the Chi-square test.

## Results

This study was carried out on 216 subjects, including 111 cases and 105 controls. These groups’ demographic characteristics are shown in Table 1. Although the BMI, WHR, total cholesterol, and triglycerides in the PCOS group were significantly higher than they were in the healthy controls (*p* <  0.05), the baseline LDL serum level did not reach statistical significance among the two groups (*p* = 0.069). As indicated in Table 1, LH/FSH (*p* <  0.0001) and testosterone (*p* <  0.0001) were statistically higher in PCOS patients compared with healthy individuals, but estrogen (*p* <  0.05) was statistically higher in the control group. Significant differences were found in the HOMA index between the healthy control and PCOS groups (*p* <  0.0001), and this factor was significantly higher in the obese group than the non-obese group (*p* <  0.001). TNF-α serum levels tended to be significantly higher in women with PCOS than controls (*p* <  0.0001).

**Table 1:** Participants’ Demographic, Anthropometric, Biochemical, and Hormonal Characteristics

Characteristic	PCOS patients (*n* = 111)	Control (*n* = 105)	*p*-value
Age (yr)	26.49 ± 6.34	27.46 ± 7.06	0.353
Height (cm)	160.59 ± 5.75	161.31 ± 5.09	0.36
Body weight (kg)	66.74 ± 14.25	62.60 ± 11.70	0.024*
BMI (kg/m^2^)	25.88 ± 5.24	24.07 ± 4.5	0.008*
WC (cm)	86.57 ± 12.78	82.14 ± 10.66	0.009*
HC (cm)	102.49 ± 12.53	100.99 ± 10.56	0.37
WHR	0.84 ± 0.1	0.81 ± 0.07	0.007*
SBP (mmHg)	105.90 ± 10.62	106.01 ± 17.38	0.55
TC (mg/dl)	187.89 ± 37.03	178.83 ± 32.79	0.05*
TG (mg/dl)	122.45 ± 24.177	105.25 ± 27.33	< 0.0001*
HDL.c (mg/dl)	41.79 ± 11.61	40.3 ± 9.96	0.377
LDL.c (mg/dl)	119.88 ± 26.86	113.67 ± 23.76	0.069
Insulin (μg/dl)	15.64 ± 5.9	8.99 ± 4.36	< 0.0001*
FBS (mg/dl)	68.83 ± 12.35	71.9 ± 14.41	0.089
HOMA-IR	2.59 ± 1.4	1.18 ± 0.99	< 0.0001*
E2 (pg/ml)	57.08 ± 38.96	73.44 ± 53.68	0.021*
Testosterone (pg/ml)	1.01 ± 0.45	0.89 ± 0.66	< 0.0001*
TNF-α (pg/ml)	2.96 ± 1.37	2.37 ± 0.94	< 0.0001*
FSH (IU/L)	7.42 ± 3.78	8.97 ± 6.62	0.005*
LH (IU/L)	10.25 ± 10.51	6.38 ± 4.44	< 0.0001*
LH/FSH	1.47 ± 1.22	0.86 ± 0.71	< 0.0001*

Data expressed as mean ± standard deviation (SD). *Abbreviations*: *BMI*, body mass index; *WC*, waist circumference; *HC*, hip circumference; *WHR*, waist hip ratio; *SBP*, systolic blood pressure; *TC*, total cholesterol; *TG*, triglyceride; *HDL*, high density lipoprotein; *LDL*, low density lipoprotein; *FBS*, fast blood sugar; *HOMA-IR*, homeostatic model assessment for insulin resistance; *E2*, estradiol; *TNF-α*, tumor necrosis factor-α; *FSH*, follicle-stimulating hormone; *LH*, luteinizing hormone, *indicates that this entity is significant statistically

### Genotype frequencies

**Fig. 2:**
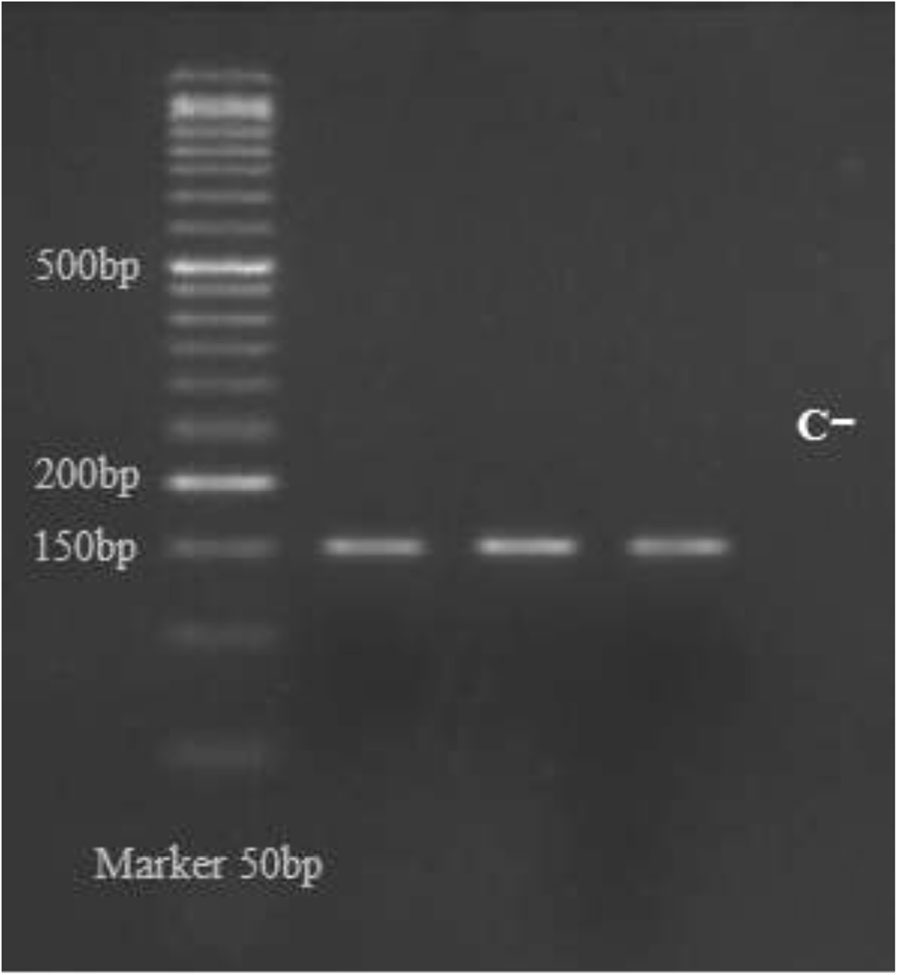
Nested PCR products gel: The gel photograph represents the amplified fragments of promoter region carrying G238A in TNF-α gene by semi nested PCR. Lane 1: 50 bp ladder size marker; lanes 2, 3, and 4: 151-bp PCR product (semi-nested PCR); Lane 5: no template control

**Fig. 3:**
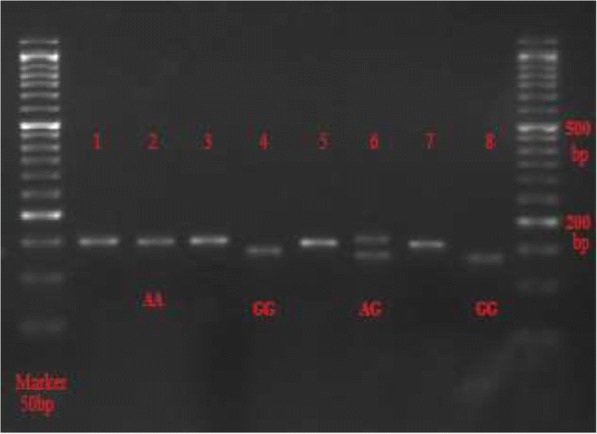
The gel photograph represents the digestion fragments with *HpaII*. Before *HpaII* digestion: Lanes 1,3, 5 and 7: 151-bp fragments. After *HpaII* digestion: Lane 2: 151-bp band representing the homozygous AA genotype (mutant type); Lanes 4 and 8: 133-bp band representing the homozygous GG genotype (wild type); Lane 6: 151- and 133-bp bands representing AG genotype (heterozygous)

Using specific primers, 151 bp of PCR products were obtained in semi-nested PCR (Fig. 2). Following digestion of PCR products with *HpaII* restriction enzyme, 151 bp, 133 bp, and 18 bp were obtained (Fig. 3).

The genotype frequencies of G238A SNP (rs361525) in PCOS and healthy controls were calculated by allele counting in the total population. As shown in Table 2, the GG genotype was found in 93.7% of PCOS patients and 91.4% of healthy individuals (*p* >  0.05), and the AG genotype was found in 5.4% of PCOS patients and 5.7% of controls (*p* >  0.05). The AA genotype was found in 0.9% of PCOS patients and 2.9% of controls (*p* >  0.05).

**Table 2:** Distribution of Genotypes and Allele Frequencies of rs361525 Polymorphism of *TNF-α* Gene in PCOS and Control Groups

TNF-α (− 238; rs361525)	Normal *n* (%)	PCOS *n* (%)	*p*-value
GG genotype	96 (91.4)	104 (93.7)	*P* > 0.05
AG genotype	6 (5.7)	6 (5.4)	*P* > 0.05
AA genotype	3 (2.9)	1 (0.9)	*P* > 0.05

*n* = Number of individuals; *TNF-α*; tumor necrosis factor-α, *PCOS*; polycystic ovary syndrome

The allelic frequencies were 0.96 and 0.04 for G and A in PCOS patients and 0.94 and 0.06 in controls respectively (not significantly different; Table 2). Thus, the A allele compared with the G allele, and vice versa, was not found to be associated with an increased risk of PCOS. The HWE was tested for the G238A SNP, but no statistically significant differences were found for this assumption (*p* <  0.05).

### Correlation analysis

Correlations between TNF-α, HOMA-IR, BMI, and triglycerides were investigated, and the results are shown in Table 3. The TNF-α serum levels were significantly correlated with the HOMA factor (*r* = 0.138, *p* <  0.05). There was a positive correlation between TNF-α serum levels and BMI, or triglyceride, but they were not significant (*p* >  0.05).

**Table 3:** Pearson Correlation Coefficients between TNF-α, HOMA-IR, BMI, and Triglyceride in the Study Subjects

Factors	TNF-α	HOMA-IR	BMI	TG
TNF-α	1	–	–	–
HOMA-IR	0.138*	1	–	–
BMI	0.050	0.444**	1	–
TG	0.113	0.263**	0.395**	1

_*Abbreviations*: *TNF-α*; tumor necrosis factor-α, *HOMA-IR*; homeostasis model assessment for insulin resistance, *BMI*; body mass index, *TG*; triglyceride, *r*;_ _correlation coefficient_

*.Correlation is significant at the 0.05 level (*p*-value)

**.Correlation is significant at the 0.01 level (*p*-value)

The HOMA factor significantly correlated with BMI (*r* = 0.444, *p* <  0.001) and triglyceride (*r* = 0.263, *p* <  0.001). BMI was positively and significantly correlated with triglyceride (*r* = 0.395, *p* < 0.001).

### Risk of PCOS and values of hormonal–biochemical factors

The association between the risk of PCOS and biochemical factors was tested with ORs and 95% CIs. The variables that were significantly associated with PCOS were considered in multiple regression analysis. As indicated in Table 4, after adjustment for confounding factors, low estrogen serum levels (OR = 0.97, 95% CI = 0.96–0.99, *p* < 0.001), high LH/FSH ratios (OR = 1.98, 95% CI =1.20–3.28, *p* < 0.01), and a high HOMA factor (OR = 5.04, 95% CI = 2.82–9.01, *p* < 0.001) were significantly associated with an increased PCOS risk. Less clear trends were observed for testosterone (OR = 1.73, 95% CI = 0.84–3.59, *p* < 0.05), BMI (OR = 0.95, 95% CI = 0.85–1.06, *p* < 0.05), and triglycerides (OR = 1.01, 95% CI = 1.00–1.02, *p* < 0.05) in this test. It seems that hyperinsulinemia and the LH/FSH ratio increase the risk of PCOS by about 5 and 2 times, respectively.

**Table 4:** Multiple Logistic Regression Analysis of Participants’ Characteristics

Characteristic	OR (Exp)	95% CI	*p*-value
Age (years)	0.95	0.88–1.02	0.17
BMI (kg/m^2^)	0.95	0.85–1.06	0.37
E2 (pg/ml)	0.97	0.96–0.99	< 0.001*
Testosterone (pg/ml)	1.73	0.84–3.59	0.13
LH/FSH	1.98	1.20–3.28	0.007*
HOMA factor	5.04	2.82–9.01	< 0.001*
TNF-α (pg/ml)	1.26	0.82–1.93	0.29
Triglycerides (mg/dl)	1.01	1.00–1.02	0.08

*Abbreviations*: *CI*, confidence interval; *OR*, odds ratio; *BMI*, body mass index; *E2*, estradiol; *HOMA-IR*, homeostasis model assessment for insulin resistance; *TNF-α*, tumor necrosis factor-α, *indicates that this intity is significant statistically

## Discussion

As an endocrine-metabolic disorder, PCOS has been demonstrated to be associated with insulin resistance, obesity, and cardiovascular disease [22–24]. Although the etiology of PCOS is not clear, but some types of cytokines, such as TNF-α, have been suggested to be related to PCOS [25].

The relationship between the rs361525 SNP of *TNF-α* polymorphism in the promoter region and PCOS has not been investigated so far, and the associations of this gene polymorphism with some hormonal and biochemical factors are not clear. However, there are a few studies regarding the effects of this *TNF-α* gene polymorphism on obesity and prediabetic individuals. Therefore, this study aimed to evaluate the relationship between the *TNF-α* gene rs361525 polymorphism and PCOS in an Iranian population. Although we found that there were significant differences in TNF-α serum levels between the two studied groups, A allele carriers compared with G allele carriers, and vice versa, were not found to be associated with an increased risk of PCOS. Populations in this study were out of the HWE; this may have been related to the small sample size, regional sample collection, inbreeding, mutation, natural selection, gene drift, gene flow, and so on.

Yu et al. [18] found that SNP rs361525 in the *TNF-α* gene was strongly associated with obesity, and G allele carriers increased the risk of obesity in Korean population. After evaluation of prediabetes and normoglycemic individuals for SNP rs361525 in the *TNF-α* gene, Dutta et al. [17] found that AA/GA genotypes were significantly more common in individuals with prediabetes, and these individuals had higher TNF-α serum levels. Progression to diabetes in these carriers was found, and a lower reversal rate after therapeutic lifestyle interventions was observed. Prediabetes is an aspect of PCOS, so TNF-α serum level results between case and control in our study is in line with this experiment. TNF-α serum levels in our study in PCOS patients compared prediabetes were lower maybe related to different type of diseases, different measurement methods, different ethnic groups.

Recent investigations have shown that rs1799964 polymorphism in the promoter region of the *TNF-α* gene could be associated with PCOS [1, 3, 16]. In contrast, several studies have revealed a lack of direct involvement of rs1800629 polymorphism of the *TNF-α* gene in PCOS patients in South Indian, Turkish, and Australian populations [2, 3, 15]. Korhonen et al. [26] indicated that rs1799724 polymorphism of the *TNF-α* gene did not have an significant association with PCOS, but a 0.17-fold increased risk of PCOS has been demonstrated in T allele carriers of this SNP.

In this study, along with the increase in serum TNF-α values, a significant increase in fasting insulin, LH/FSH ratios, testosterone, cholesterol, triglyceride, and BMI were observed in PCOS patients compared with the controls. Wherever the serum level of TNF-α increased, elevation of the HOMA factor was observed, with a positive correlation. In contrast, an increase of the LH/FSH ratio and HOMA-IR contributed to the progression of PCOS. This result means that TNF-α could indirectly exacerbate PCOS, and it illustrates the importance of serum TNF-α levels, LH/FSH ratios, and HOMA-IR in PCOS diagnosis.

Consistent with our results, Xiong et al. [20] showed that higher serum triglyceride and TNF-α in PCOS patients represented the main cause of low-grade chronic inflammation. Moreover, Pawelczak et al. [19] demonstrated that free testosterone and serum TNF-α were elevated in adolescents with PCOS. It is interesting that the serum TNF-α values were different among various studies. As in our study, fluctuations in the TNF-α serum level in previous research may have been related to variations in hormonal regulation among different subjects, inflammation-mediated synthesis mechanisms, the length of time since PCOS diagnosis, and different hereditary and genetic backgrounds [26].

Consistent with our results, Gao et al. [27] found that TNF-α and the HOMA index were higher in women with PCOS. Accordingly, it has been demonstrated that TNF-α may induce insulin resistance by serine phosphorylation in IRS-1 [9, 11]. Samy et al. [28] reported that such inflammatory markers correlated significantly with the BMI and HOMA index in PCOS patients. It can be postulated that TNF-α and HOMA index may be prognostic and diagnostic factors [27].

There have been reports that the index of insulin sensitivity is inversely correlated with circulating TNF-α, interleukin-6 (IL-6), and C-reactive protein (CRP) levels. Therefore, inflammatory cytokines may induce insulin resistance. Because chronic inflammatory markers enhance insulin resistance and hyperandrogenism, therefore they are involved in the pathogenesis of PCOS [29, 30]. Accordingly, it has been shown that TNF-α facilitates the effects of insulin and insulin-like growth factor 1 (IGF-I) on the ovary, thereby stimulating proliferation and steroid genesis in rat theca cells in vitro [29].

Choi et al. [13] assessed the hormonal and biochemical profiles and TNF-α serum level as an inflammatory cytokine in non-obese PCOS patients in a Korean population. When women with PCOS were divided into those with and without hyperandrogenism, the TNF-α serum level was significantly higher among women with PCOS compared with controls, as well as in the hyperandrogenism group compared with those without hyperandrogenism. In another study, it was shown that serum TNF-α, free and total testosterone, androstenedione, and dehydroepiandrosterone (DHEA) were elevated in PCOS patients compared with the control group [14]. Likewise; testosterone and TNF-α levels were higher in PCOS patients compared with the control group in our study.

## Conclusions

Despite a lack of significant difference in the rs361525 polymorphism of the *TNF-α* gene between PCOS and normal individuals, the serum level of TNF-α is increased in PCOS patients and positively correlates with the HOMA factor. In addition, the LH/FSH ratio and HOMA factor increase the risk of PCOS. Therefore, TNF-α could indirectly contribute to PCOS progression.

## Abbreviations

BMI: Body mass index
CI: Confidence interval
CRP: C-reactive protein
E2: Estradiol
ELISA: Enzyme-linked immunosorbent assay
FSH: Follicle-stimulating hormone
HOMA-IR: Homeostasis model assessment for insulin resistance
HWE: Hardy–Weinberg equilibrium
IGF-I: Insulin-like growth factor 1
IL-6: Interleukin-6
IRS-1: Insulin receptor substrate-1
LH: luteinizing hormone
OD: Optical density ratios
OR: Odds ratio
PCOS: Polycystic ovary syndrome
PCR-RFLP: Polymerase chain reaction- restriction fragment length polymorphism
SD: Standard deviation
SNP: Single-nucleotide polymorphism
TNF-α: Tumor necrosis factor-α
WHO: World Health Organization
WHR: Waist–hip ratio
